# Data for dynamics analysis of riverine dissolved organic in watersheds

**DOI:** 10.1016/j.dib.2018.08.171

**Published:** 2018-09-06

**Authors:** Fabien Rizinjirabake, Abdulhakim M. Abdi, David E. Tenenbaum, Petter Pilesjö

**Affiliations:** a Lund University, Department of Physical Geography and Ecosystem Science, Geocentrum 2, Sölvegatan 12, 22362 Lund, Sweden.; b University of Rwanda, College of Science and Technology, Biology Department, Rwanda

## Abstract

This data article presents water stage, flow, and net primary productivity (NPP) data that were used to analyze the dynamics of the riverine dissolved organic carbon (DOC) dynamics in the Rukarara River watershed in Rwanda. We measured water stage data every 15 min and calculated daily averages used to estimate flow based on rating curves. The rating curves were produced using several measured contemporaneous water stage and flow data. Estimated flow data were used to calculate water dissolved organic carbon (DOC) loadings separate the total stream flow into quick and baseflow. Annual NPP data for a 15-year period were used to estimate the effect of proportion of stream DOC loading on carbon sequestration within the Rukarara River watershed.

**Specifications table**

**Table:** 

Subject area	Eco-hydrology
More specific subject area	The subject deals with effects of hydrological processes on the distribution, structure, and function of ecosystems, and the biotic processes on elements of the water cycle. Eco-hydrology has main applications in water resource management, ecosystem restoration and conservation.
Type of data	Tables
How data was acquired	Field measurements by PT2X pressure transducer flow gauges with integrated data logger, MiniDiver and current meters
Data format	Analyzed
Experimental factors	Stream DOC samples were transported to the laboratory on ice and 1 ml of sulfuric acid (H_2_SO_4_) was always added to samples to reduce the microbial activity
Experimental features	Water stage data were measured with the PT2X and Minidivers sensors, whereas current data were measured with mechanical current meters. Stream DOC data were analyzed on TOC analyzer.
Data source location	Rukarara River watershed (29°15–29°35E and 2°20N-2°35 S)
Data accessibility	Data is with this article
Related research article	Rizinjirabake, F., Abdi, A. M., Tenenbaum, D. E., & Pilesjö, P. (2018). Riverine dissolved organic carbon in Rukarara River Watershed, Rwanda. *Science of the Total Environment*, *643*, 793–806. https://doi.org/10.1016/j.scitotenv.2018.06.194.

**Value of the data**

• Provided high resolution temporal data of stream water stage in a tropical watershed for a two-year period
• Provided daily water flow data of streams in a tropical watershed for a two-year period
• Provided stream dissolved organic carbon data in tropical environment
• Used MODIS NPP data for carbon dynamics analysis

## 1 Data

We present water stage and flow data, stream DOC concentration for the Rukarara River and its three tributaries for a period from March 2015 to February 2017. We also present NPP data (spatial resolution: 0.500 km) for an agriculture and forest mixed tropical watershed. Our data were used to characterize the dynamics of DOC in the Rukarara River watershed, but can also be used, for example, for studying the dynamics of other important dissolved nutrients (e.g nitrogen, phosphorus, etc) for studying the function of both aquatic and terrestrial ecosystems. Our data provide a valuable resource for future research on potential effect of environmental change on dissolved organic matter in the watershed. This is important for mitigation and restoration strategies for a better management of the watershed under ongoing global environmental change.

## 2 Experimental design, materials, and methods

### 2.1 Stage data

We collected data stream stage in gauging stations that were installed at three streams alongside the large river outlet. In total, four different sites were used to collect water stage data, namely natural forest, tea plantations, farm and outlet locations. Sites were located at straight stream and river reaches with a channel bank that is relatively uniform. At each gauging station, water stage data were recorded by automatic pressure transducers and mini-divers. Both instruments are reliable autonomous measuring and recording of water stage. Transducers and mini-divers were installed in ad hoc iron stilling wells installed, fixed in place with concrete and closed with padlocks. Stage data were recorded as a function of pressure, every 15 min. Table 1 presents the mean daily stage data for the period from March 2015 to February 2017.

### 2.2 Measured flow data

Flow data were calculated by multiplying the average stream flow velocity times the cross-sectional area of the stream. Velocity data were collected several times at the four sites during the period from October 2016 to February 2017. Velocity data were collected by the Six-Tenths-Depth method [2] using a small size current meter for the farm site and medium size current meter for the three remaining sites. Section areas were designed in the sense recommended by Baldassarre and Montanari (2009) : the volume of flow in each subsection should be less than 5% of the total volume and the difference in current values between two adjacent points should not be more than 20% of the higher value (Baldassare and Montanari, 2009) [1]. Distance between two successive verticals was 0.5 m for the outlet of the large river, 0.40 m for tea and natural forest streams sites, and 0.10 m for the farm. The current for the river or the stream was produced by summation of current values of all verticals of interested stream. Table 2 presents measured and calculated flow data (m^3^/s) per site, and data accuracy as calculated evaluated using the Nash-Sutcliffe efficiency (NSE) [4].

### 2.3 Calculated flow data

Rating curves were produced based on measured contemporaneous stage and current data. The rating curves performed well (0.81 ≤ R^2^ ≤ 0.91) and therefore were used in calculating flow data. Table 3 presents the calculated flow data (m^3^), their range, mean absolute deviation and standard deviation.

### 2.4 Stream DOC data

We collected stream water from which DOC concentrations (mg/L) were analyzed. Sampling sites were located in natural forest, tea plantation, and farm sites, and at the outlet of the large river. The sampling sites were selected at straight river reaches with a channel bank that is relatively uniform. The samples were analyzed using TOC analyzer (the Sievers InnovOx Laboratory TOC Analyzer) at the chemistry laboratory of the College of Science of University of Rwanda, Huye Campus. Before analysis, samples were filtered using nylon membrane filters of a pore size of 0.80 µm and 0.45 µm. Table 4 presents stream DOC data for the sampling period, and precision measures including range, mean absolute deviation and standard deviation.

### 2.5 Net primary productivity data

The paper presents net primary productivity (NPP) data (spatial resolution: 0.5 km) covering 15 years, from 2000 to 2014. Data were provided by the NASA LP DAAC, a center of the NASA Earth Observation System (EOS) program that provides continuous estimates of NPP across Earth׳s entire vegetated land surface. The NPP data are calculated using the MOD17 Algorithm, based on the logic of light use efficiency (LUE) [3]. Table 5 summarizes annual NPP data for the period from 2000 to 2014 and presents their range, mean absolute deviation and standard deviation.

## References

[1] Di Baldassarre G., Montanari A. (2009). Uncertainty in river discharge observations: a quantitative analysis. Hydrol. Earth Syst. Sci..

[2] S.L. Dingman, Fluvial hydrology, 1984.

[3] Monteith J.L. (1972). Solar radiation and productivity in tropical ecosystems. J. Appl. Ecol..

[4] J.E. Nash, J.V. Sutcliffe, River flow forecasting through conceptual models part I—a discussion of principles, J. Hydrol. 10 (3) (1970) 282–290 10.1016/0022-1694(70)90255-6.

